# Targeted Therapy After Brain Radiotherapy for BRAF-Mutated Melanoma With Extensive Ependymal Disease With Prolonged Survival: Case Report and Review of the Literature

**DOI:** 10.3389/fonc.2019.00168

**Published:** 2019-03-26

**Authors:** Ibrahim Abu-Gheida, Samuel Chao, Erin Murphy, John Suh, Glen H. Stevens, Alireza M. Mohammadi, Michael McNamara, Jennifer S. Yu

**Affiliations:** ^1^Department of Radiation Oncology, Taussig Cancer Center Institute, Cleveland Clinic, Cleveland, OH, United States; ^2^Department of Neurology, Neurological Institute, Cleveland Clinic, Cleveland, OH, United States; ^3^Department of Neurological Surgery, Neurological Institute, Cleveland Clinic, Cleveland, OH, United States; ^4^Department of Hematology and Medical Oncology, Taussig Cancer Center Institute, Cleveland Clinic, Cleveland, OH, United States; ^5^Department of Cancer Biology, Lerner Research Institute, Cleveland Clinic, Cleveland, OH, United States

**Keywords:** melanoma, ependymal disease, leptomeningeal disease, whole brain radiation, radiosurgery, BRAF inhibitor, MEK inhibitor, brain metastasis

## Abstract

Melanoma brain metastasis with ependymal spread/metastases is uncommon. These cases are frequently classified together with leptomeningeal disease. However, the commonalities and differences in the underlying pathophysiology and clinical outcomes between these two types of spread are not clear. Very few reports on long term outcome and durable central nervous system (CNS) disease control have been reported in the literature. Here, we report a case of a 45 year-old Caucasian lady with BRAF-V600E mutant metastatic melanoma to the brain who had whole brain radiotherapy followed by two Gamma knife radiosurgery treatments for localized disease progression. She then developed extensive ependymal disease progression with no evidence of leptomeningeal spread. She was treated with a repeat course of whole brain radiotherapy and maintained on BRAF and MEK inhibitors with durable CNS disease control for more than a year. This study reviews the management of BRAF-V600E mutant melanoma with ependymal involvement. Management using radiation therapy with maintenance targeted therapy seems to be a reasonable approach to this challenging disease.

## Introduction

**Figure 1:**
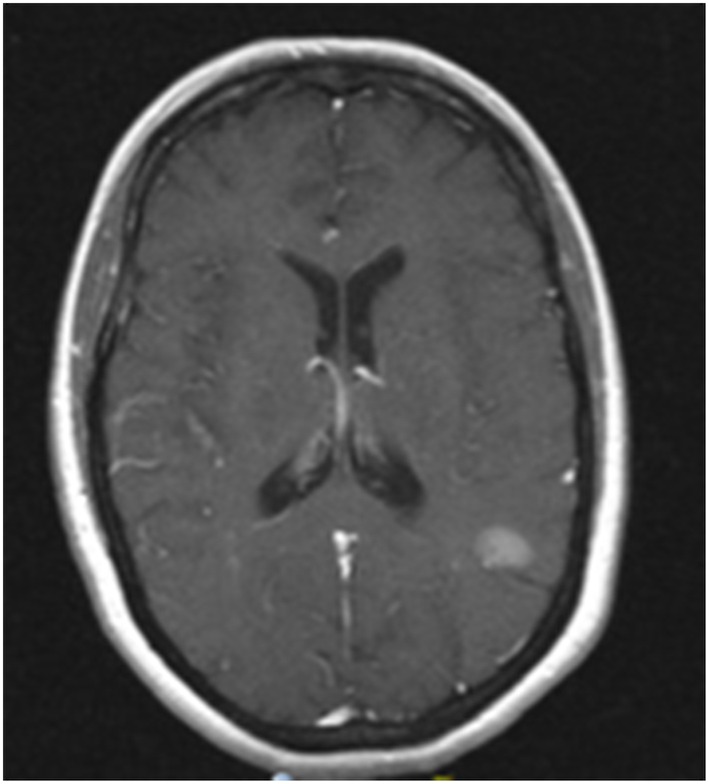
MRI image of progression of left temporoparietal metastasis. Contrast enhanced T1 MRI showing a 1.6 cm left temporoparietal lesion that increased in size after completion of whole brain radiation. This lesion was treated subsequently with stereotactic radiosurgery.

**Figure 2:**
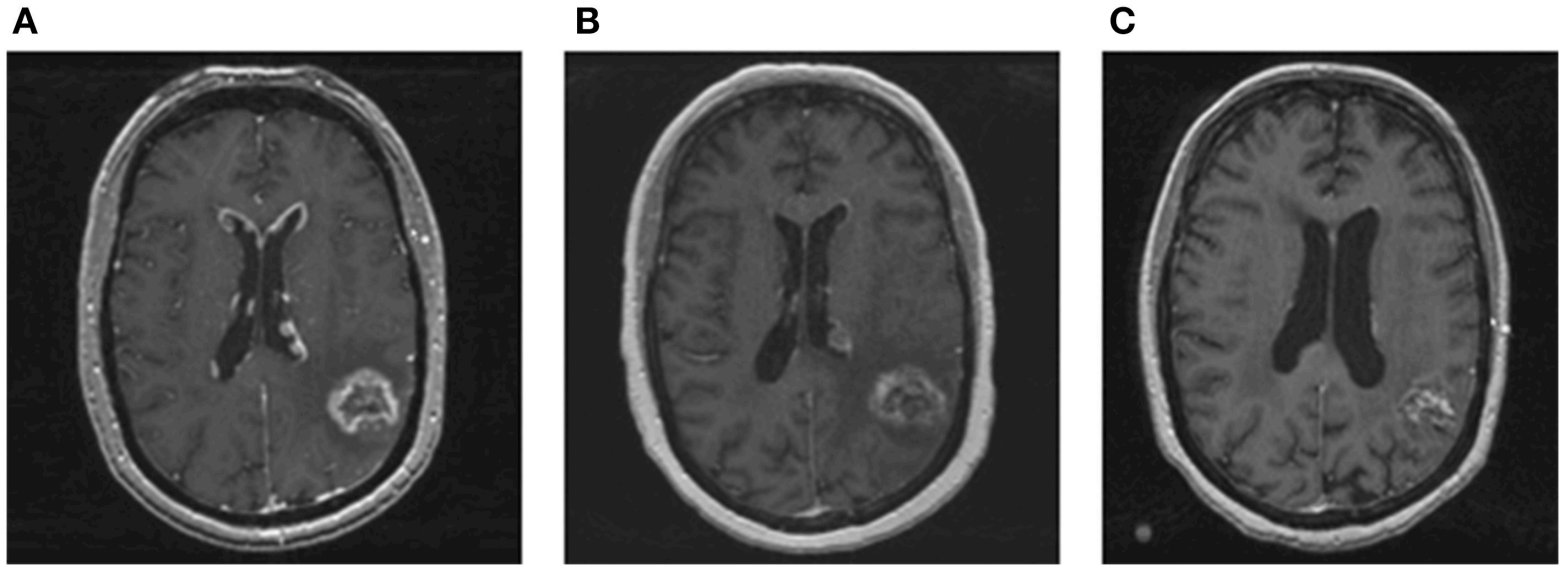
MRI images of ependymal disease and response to whole brain re-irradiation. **(A)** Contrast enhanced T1 MRI showing new enhancing, linear, and nodular lesions along the ventricles consistent with ependymal disease. **(B)** Contrast enhanced T1 MRI performed 2 months after whole brain re-irradiation showing a significant and rapid decrease in ependymal enhancement. The treated metastasis in the left temporoparietal area was stable. **(C)** Contrast enhanced T1 MRI performed 1 year after whole brain radiation showing resolution of nodular lesions along the ventricles and no new brain metastases.

A 45 year-old Caucasian woman presented to our institution for management of brain metastasis. Four years prior to her presentation, she was diagnosed with left upper back BRAF V600E mutant melanoma. She underwent wide local excision with sentinel lymph node biopsy of the left axilla with no nodes positive. She did well for the next 3 years until she presented with an isolated biopsy-proven right axillary recurrence. She underwent right axillary lymph node dissection with pathology demonstrating one out of 16 lymph nodes positive, measuring 3.5 cm with extranodal extension. The patient then received adjuvant radiation to the right axilla followed by interferon treatment for 1 year. At the end of interferon treatment, surveillance PET/CT showed no evidence of extracranial systemic disease. However, MRI brain showed a solitary 1.6 cm left temporoparietal lesion (Figure 1). The patient was asymptomatic and was treated with whole brain radiation therapy (WBRT) at an outside hospital. Two months after completing radiation, the patient developed dizziness and had an episode of generalized tonic clonic seizure. MRI brain showed progression of the left temporoparietal lesion. She was referred to our center for further treatment and was subsequently treated by Gamma Knife radiosurgery (GKRS). Follow up MRI showed that the treated lesion was stable, but there was an additional enhancing lesion in the left inferomedial cerebellum. This lesion was asymptomatic. The patient also received GKRS to the cerebellar lesion with excellent control. Serial brain MRIs and MRI perfusion over the next 6 months showed no new lesions. The patient developed radiographic evidence of radiation necrosis in the left parietal lobe and was maintained on steroids with good response. However, she developed side effects from steroids including proximal muscle weakness and significant weight gain that interfered with her functioning. Attempts to wean her off steroids were unsuccessful. She was being evaluated for laser ablation to reduce cerebral edema associated with her radiation necrosis. On workup for this procedure, she was found to have progression of brain metastases including a new 3 mm enhancing focus involving the right anterior body of corpus callosum and enhancing, linear and nodular lesions along the ventricles consistent with ependymal spread (Figure 2A). Complete spine MR imaging showed no evidence of gross spinal leptomeningeal disease. Considering her extensive brain disease, she was not a candidate for laser ablation. She received a second course of WBRT. She then received dual BRAF/MEK inhibition with dabrafenib and trametinib for 1 year. MRI 2 months post-WBRT showed a decrease in ependymal enhancement; the previously treated metastasis was stable (Figure 2B). In the meantime, multiple failed attempts were made to wean the patient off of steroids. The patient suffered from steroid related side effects including weight gain, diabetes, and proximal muscle atrophy and was switched to bevacizumab for radiation necrosis. Bevacizumab was discontinued after the patient developed gastrointestinal bleeding. During this time, she continued to be maintained on dabrafenib and trametinib. Her last follow up brain MRI showed slight increase size of the left parietal radiation necrosis, but her ependymal disease was stable and there were no new lesions (Figure 2C). Within 1 month, however, the patient developed widespread disease. She enrolled in hospice care and passed away 1 week later.

## Background

The incidence of melanoma has been rising for the last 30 years. It is estimated that ~76,000 individuals will be diagnosed with melanoma and about 10,000 will succumb to their disease (1). Being the third most common brain metastasis diagnosed after lung and breast cancer, melanoma has the highest risk of spread to the CNS among all common cancer types with a 40–60% chance of patients developing brain metastases at some point in the course of their disease. Autopsy series identified CNS involvement in up to 80% of patients with metastatic melanoma (2). Several studies indicate a median survival of ~4 months once patients are diagnosed with melanoma brain metastasis (2, 3). Poor non-CNS disease control, poor performance status, leptomeningeal disease (LMD) defined as the infiltration of cancer cells in pia mater and arachnoid membrane, more than three CNS brain metastatic melanoma lesions, and a higher cumulative intracranial tumor volume have been associated with poor survival (2, 4, 5). Surgery for melanoma brain metastasis with or without adjuvant whole brain radiation therapy has been shown to improve the median survival to ~9 months regardless whether patients have single or multiple brain metastases (3). However, depending on the location and the morbidity of surgery, this treatment option is sometimes not reasonable, especially in the setting of diffuse leptomeningeal involvement or periventricular ependymal involvement.

Ependymal spread is rare and is frequently associated with LMD. Flow of tumor cells in cerebrovascular fluid (CSF) through the ventricular system can seed the leptomeninges. Conversely, LMD along the surface of the brain and spinal cord may circulate through the CSF and deposit tumor cells along the ependymal surface leading to periventricular ependymal disease involvement. Ependymal disease is often clustered with LMD, although it is unclear whether differences exist in the underlying biology of and clinical outcomes between these two different presentations (6).

BRAFV600E mutation is observed in 40–50% of melanomas (7). The incidence of brain metastasis in BRAF-mutated or BRAF-wild type tumors is similar. However, a lower incidence of brain metastases was observed in patients with BRAF-mutated tumors who received targeted therapy before the development of brain metastasis, suggesting that targeting BRAF may reduce metastatic spread to the brain or inhibit the growth of micrometastatic brain metastases (8). BRAF mutated melanoma brain metastases were shown to be more resistant to conventional treatment (including systemic agents alone, WBRT, or radiosurgery) when compared to BRAF wild type metastases (HR 2.83 *p* = 0.01) (9). LMD is particularly associated with poor median survival of only 2 months despite treatment. Little is known about ependymal spread without LMD. Our patient with BRAFV600E mutant metastatic melanoma had extensive ependymal spread with no evidence of gross LMD. She survived for 28 months after initial diagnosis of brain metastasis and 11 months after diagnosis of significant ependymal disease.

## Discussion

Targeted therapy has modernized the treatment approach to BRAF-mutant metastatic melanoma. Vemurafenib was shown in a randomized controlled trial to prolong overall survival (10). Dabrafenib has been shown to improve progression-free survival (11). Single-agent BRAF inhibitors showed an excellent systemic response rate of ~50%. Response duration, however, was short lived with median of 6–7 months (10, 11). Both of these drugs are known to be effluxed by the intact blood brain barrier's p-glycoprotein hence have a poor CNS penetrance (12). Dabrafenib has been shown to have slightly better CNS penetration than vemurafenib (13). Clinically, the intracranial tumor response to vemurafenib in BRAF-mutation-positive melanoma patients was seen in seven out of 19 patients (37%) (14). The intracranial response to dabrafenib ranges between 31 and 39% in patients with brain metastatic BRAF mutated melanoma from the BREAK –MB phase two trial (15). Addition of MEK inhibitors to dabrafenib can improve the rate and duration of response (16). Combination of BRAF and MEK inhibitors are now the standard of care for BRAF-mutated melanoma patients and have demonstrated some intracranial activity. Objective response rates are as high as 31% with dabrafenib in the BRAF mutant population (17, 18). Despite that, given the relatively poor BBB penetrance of anti-BRAF drugs, the brain remains a common site of treatment failure for BRAF-targeted therapy driving the poor outcome and survival of this patient population (19). Moreover, LMD has been traditionally approached by best supportive care and palliative therapy given its very poor outcome despite the multiple treatment modalities utilized (2).

However, in our case, we present a patient who survived 11 months after being diagnosed with significant ependymal disease. This was remarkable compared to a median survival of 8–10 weeks from the literature (2, 20). Unfortunately, this patient ultimately succumbed to her disease and developed steroid dependent radiation necrosis along with associated side effects. Similar case reports reported long term survival outcome in patients with BRAF-mutated melanoma with LMD (21). Some case reports have demonstrated improvement in LMD after vemurafenib (22, 23). Interestingly, we noted that the longest median survival was attained in patients maintained on combination BRAF/MEK inhibitors after brain irradiation (21, 23). Combination treatment is needed as patients with symptomatic brain metastases need a rapid response to relieve symptoms which is attained by radiation. Maintenance BRAF/MEK inhibitors are needed to sustain the response. A recent report of 39 patients with melanoma with LMD showed mixed response to radiation and targeted therapy. The majority of patients had an extremely poor prognosis, as expected, however, targeted therapy helped to maintain CNS disease remission especially when used in combination with radiation therapy (24).

Our patient,despite having nodular ependymal disease, showed impressive brain control and overall survival after radiation and targeted therapy. Interestingly, she did not show evidence of gross disease involving her leptomeninges. Whether she had circulating tumor cells in her CSF is unclear. The course of disease in our patient suggests that ependymal disease does not necessarily lead to LMD. This study suggests that patients with ependymal disease in the absence of LMD may have a different natural history than those with LMD and therefore should be classified separately. Moreover, aggressive therapy with radiation and targeted therapy may offer relatively long-term disease control.

## Concluding Remarks

Metastatic melanoma with spread to the ependymal lining or leptomeninges is associated with very poor outcomes. Ependymal disease does not necessarily lead to LMD and may have more favorable outcomes. Our patient with BRAF-mutant melanoma survived for 11 months after diagnosis of nodular ependymal spread. Aggressive multimodality treatment with radiation therapy and targeted therapy should be considered for these patients.

## Ethics Statement

Written consent from the patient's next of kin was obtained for publication of the case report.

## Author Contributions

IA-G summarizing the case and writing the paper. GHS, SC, EM, JS, AM, and MM reviewing and editing manuscript. JY revising, editing, and coordinating the manuscript.

### Conflict of Interest Statement

The authors declare that the research was conducted in the absence of any commercial or financial relationships that could be construed as a potential conflict of interest.
